# Evaluating the effect of apex position and rocker in curved rocker shoes

**DOI:** 10.1186/1757-1146-7-S1-A66

**Published:** 2014-04-08

**Authors:** Jonathan D Chapman, Stephen J Preece, Christopher J Nester, Bjoern Braunstein, Angela Höhne, Gert-Peter Brüggermann

**Affiliations:** 1 School of Health, Sport and Rehabilitation Sciences, University of Salford, UK; 2 Institute of Biomechanics and Orthopaedics, German Sport University, Cologne, Germany

## Background

Curved rocker shoes are designed with a contoured outsole which can be characteristics by three principle design features: rocker angle, apex angle and apex position. Although these shoes are routinely prescribed to reduce in-shoe pressure in patients with diabetes, there is only minimal scientific evidence to inform the choice of value for each of the three design features. Results from a previous study [1], suggested that a 95° apex angle may be the best compromise for offloading the different regions of the forefoot. The results of this study also suggested that higher rocker angle may lead to decrease pressure, however, this study did not quantify the precise effect of rocker angle and apex position in shoes with a 95° apex angle.

## Objective

To investigate the combined effect of varying rocker angle and apex position in rocker shoes designed with a 95° apex angle. A relatively thick outsole is required to produce a rocker shoes with a rocker angle of 20° or greater. Therefore we also sought to quantify the proportion of individuals with diabetes for which it would be possible to achieve acceptable pressure offloading with a 15° rocker angle.

## Methods

A factorial design was used to investigate the effect of the two design features: rocker angle (15° or 20°) and apex position (52%, 57%, 62%, 67% shoe length). Eight rocker shoes were manufactured to cover this range of design features and tested on n=87 patients with diabetes. Each participant walked in every shoe at 1 ms^-1^ whilst in-shoe pressure was recorded. A two-way ANOVA test was used to understand the main effect of each of the design features on peak plantar pressure and also to identify any possible interactions. A threshold of 200 KPa [2] was then used to identify individuals who did not experience sufficient offloading with a 15° rocker angle.

## Results

With both the 15° and 20° rocker angle the mean optimal apex position was found to be 52% of shoe length. Furthermore, there was a significant reduction in peak pressure when rocker angle was increase from 15 to 20°. Despite this increase, 66% of participants experienced sufficient offloading with a 15° rocker angle provided an optimal apex position was selected.

## Conclusion

Increasing rocker angle decreased peak plantar pressure. However, provided the optimal apex position of 52% shoe length was selected, it was possible to achieve acceptable offloading with a 15° rocker for a large proportion of individuals.
